# Acute Appendicitis Secondary to Appendiceal Endometriosis

**DOI:** 10.1155/2020/8813184

**Published:** 2020-10-10

**Authors:** João Paulo Nunes Drumond, André Luis Alves de Melo, Demétrius Eduardo Germini, Alexander Charles Morrell

**Affiliations:** Rede D'OR São Luiz, São Paulo, Brazil

## Abstract

Endometriosis in the vermiform appendix is a rare condition that affects women of childbearing age. The clinical picture can simulate inflammatory acute abdominal pain, especially acute appendicitis. Laboratory and imaging tests may assist in the diagnosis but are not conclusive. This article reports a case of acute appendicitis caused by appendiceal endometriosis for which laparoscopic appendectomy and diagnostic confirmation were performed after histopathological analysis.

## 1. Introduction

Acute appendicitis is the inflammation of the mucosa of the vermiform appendix, which progresses to its most external parts; it represents the most common emergency surgical condition [1]. In most cases, the pathophysiology of acute appendicitis is caused by luminal obstruction, which leads to increased pressure, bacterial proliferation, venous congestion, and mucosal ischaemia and may cause perforation of the organ. Appendiceal obstruction may occur due to internal obstructive causes, such as the presence of fecaliths, lymphoid hyperplasia, parasites, or neoplasms [2].

More than 300,000 appendectomies are performed every year in the United States [2]. In Brazil, 126,280 appendectomies were performed in 2019, of which 117,913 were performed by conventional surgery, and 8,367 were performed by videolaparoscopy [3].

Endometriosis is the presence of extrauterine endometrial tissue, and it affects 6 to 10% of women of childbearing age. In 3 to 37% of cases, it affects the gastrointestinal tract [4]. Deep endometriosis occurs when there is a subserosal or subperitoneal invasion greater than 5 mm; it is a frequent and severe presentation of endometriosis. Most cases of deep intestinal endometriosis invade the rectum and sigmoid colon and are usually multifocal [5]. However, appendiceal endometriosis is a rare phenomenon, with a reported incidence in the literature ranging from 0.05% to 1.69% [6–11] of patients with endometriosis. Among patients who are surgically treated for deep endometriosis, appendiceal involvement is observed in 2.6% to 13.2% of cases [12, 13].

In symptomatic cases, this condition can simulate acute appendicitis [6, 7, 14]. Preoperative diagnosis based on physical examination and imaging tests can be challenging. Nonetheless, this condition should be considered in the differential diagnosis of acute abdominal pain located in the right lower quadrant in women of childbearing age [7, 14].

The clinical picture may be characterized by lower abdominal pain and lower back pain on the right [6, 8, 15]; pain in the right iliac fossa [7]; or, even more characteristic of acute appendicitis, periumbilical pain migrating to the right iliac fossa [14, 16]. Associated symptoms such as anorexia, nausea, and vomiting are frequently present [8, 14–16]. Signs of peritonitis [6–8] and changes in vital signs, such as fever and tachycardia, were not found [7, 8, 14], except in one report of painful abrupt positive decompression located at McBurney's point [16].

In the laboratory evaluation, leucocytosis or increased C-reactive protein were frequent findings [7, 8, 14–16]. Abdominal ultrasound (US) may not identify the vermiform appendix [7]; evidence of dilated and noncompressible appendix, suggestive of acute inflammation [14, 16]; or a homogeneous hypoechoic tubular lesion with thickened walls located in the pericaecal area [7]. In general, abdominal US may show signs of inflammation or luminal obstruction, and in transvaginal US with colon preparation, hypoechoic nodular lesions or irregular thickening of the appendix wall may appear [5].

Computed tomography may not show changes [14]; it may show only thickening of the appendiceal wall [8], with or without a surrounding hypodense masses [5]; or it may show a mass inside the appendix [6], parietal thickening, and an intraluminal mass with periappendiceal infiltrate [15] or even focal nodules in the appendix body [5].

The presence of endometrial tissue in the caecal appendix is the basis for the diagnosis of appendiceal endometriosis, and it is confirmed by the histopathological analysis of the specimen [6–8, 14]. Laparoscopy is always the first surgical option [7, 8, 14, 15]; however, depending on the findings, conversion to open surgery may be necessary [8, 15]. Endoscopic biopsy may be recommended, but with the possibility of an inconclusive anatomopathological result [6]; or midline infraumbilical laparotomy may be indicated at the very beginning [16].

Histopathological analysis discards the diagnosis of acute inflammatory processes in the appendix with the absence of polymorphonuclear infiltrate [8, 14]; however, an increase in the number of lymphoid follicles can occur [16]. Normal mucosa is present but with clusters of glands and endometrial stroma in the serosa and macrophage infiltrate with haemosiderin inclusions in the muscle layer [8, 14, 16]. A mass of endometrial stromal and glandular tissue can also be observed that involves the appendix and mesoappendix [7] and has intraluminal haemorrhage foci [7, 8]. Larger nodular lesions can be found, including lumen obliteration and secondary mucocele formation, and can invade adjacent structures [15].

The absence of dysplasia or malignancies is also a frequent finding, but up to 13% of cases of appendiceal endometriosis may have intestinal metaplasia, especially when there is marked distortion of the appendix, mass formation, and luminal obliteration [6, 7, 14, 15]. These findings generate doubt regarding the diagnosis of mucinous neoplasia or carcinoid tumour [17]; this is the most common neoplasm of the caecal appendix, with an incidence of up to 0.32% in appendectomy specimens [18].

This article is aimed at reporting a case of acute appendicitis caused by endometriosis of the caecal appendix, which was diagnosed after laparoscopic appendectomy and confirmed by histopathological analysis. The literature review was based on a *PubMed* search of articles published in the last 5 years.

## 2. Case Presentation

The patient was 32 years old and was previously healthy. The patient presented at the emergency room in September 2019 was complaining of abdominal pain over the last 24 hours, which began in the epigastrium and migrated to the right iliac fossa. Anorexia, nausea, and vomiting were associated symptoms. She denied fever, diarrhoea, dysmenorrhea, irregular menstrual cycle, or other symptoms. There were no factors that worsened or improved the pain. Vital signs at admission: blood pressure 120/70 mmHg, afebrile, and heart rate 70 bpm. On physical examination: good general condition, lucid and oriented, rosy cheeks, hydrated, and with normal breathing. Abdomen flat and flaccid, tender in the right iliac fossa, normal bowel sounds, with involuntary guarding and no signs of peritoneal irritation. Rovsing and Blumberg signs were negative.

**Figure 1:**
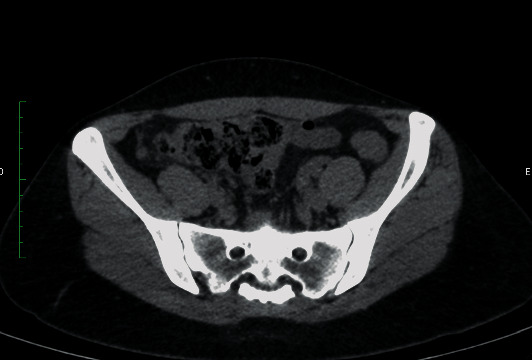
Computed tomography—axial section.

**Figure 2:**
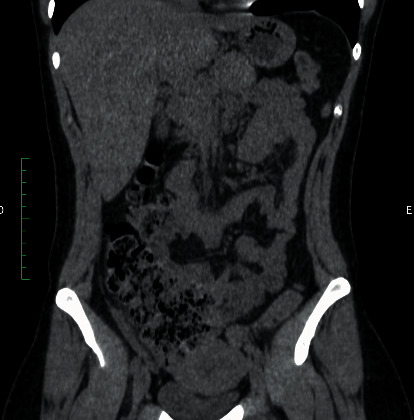
Computed tomography—coronal section.

Given the diagnostic hypothesis of acute abdomen, complementary tests were requested. Of the laboratory parameters analysed, leucocytosis of 18,200/mm [3] was observed. The CT scan of the abdomen and pelvis without contrast (Figures 1 and 2) revealed a retrocaecal appendix with an increased diameter (0.8 cm) and densification of adjacent adipose planes, without the presence of collections, free fluid, or pneumoperitoneum, which suggests acute appendicitis.

Once the diagnosis was made, the patient was hospitalized and submitted to fasting and antibiotic prophylactic (ceftriaxone 1 gram and metronidazole 500 mg); she underwent a laparoscopic appendectomy without complications, after 8 hours admission to Emergency Room. During the intraoperative period, we observed a caecal appendix compatible with acute appendicitis with hyperaemia, oedema, and adjacent fibrinous exudate. The abdominal inventory did not show findings in ovaries and uterus or any evidence of extra pelvic endometriosis. The patient progressed well and was discharged on the 1st postoperative day; she was prescribed with ciprofloxacin 500 mg every 12 hours for 7 days, nimesulide 100 mg every 12 hours for 3 days, metamizole 500 mg every 6 hours for pain, ondansetron 8 mg in case of nausea or vomiting, and simethicone 25 drops every 8 hours for abdominal discomfort. She was instructed to present to an outpatient service in 10 days for follow-up.

**Figure 3:**
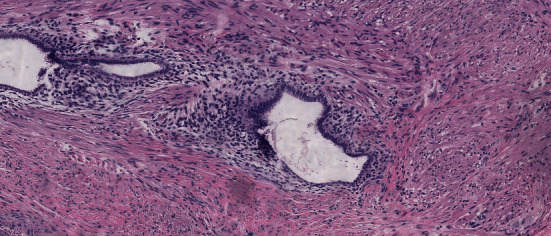
Magnification 100×.

**Figure 4:**
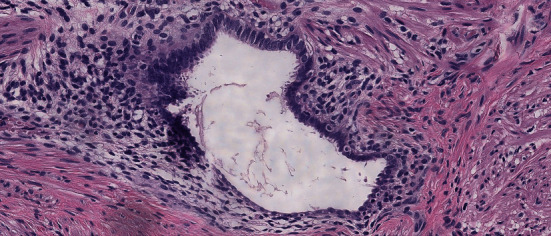
Magnification 200×.

The macroscopic histopathological analysis revealed a caecal appendix 8.1 cm long and 0.4 cm wide with velvety and serous mucosa that were greyish-brown in colour, without additional findings. Microscopy (Figures 3 and 4) showed acute suppurative appendicitis associated with endometriosis of the stromal and glandular pattern involving the muscularis propria layer, with acute serositis. No dysplasias were noted.

## 3. Discussion

This reported case had a history, physical examination, laboratory, and imaging tests compatible with acute appendicitis. The anatomopathological analysis confirmed the diagnosis and added new data, and appendiceal endometriosis was the probable aetiology of acute inflammation.

The pain initiating in the epigastric region with migration to the right iliac fossa and the associated symptoms are consistent with the reports of two other authors [14, 16], as were the presence of leucocytosis, a finding that is highly frequent [7, 8, 14–16].

Some authors [6, 7, 14] state that acute appendicitis is the most common manifestation of appendiceal endometriosis, but the incidence of this event is not fully understood. Other manifestations include mild, acute, or chronic pain in the right lower quadrant, intestinal obstruction, intussusception, melena, or intestinal perforation [5]. Other authors [12, 13] have associated it with deep endometriosis, specifically with involvement of the pelvis, bladder, caecum, and ileum, and with adenomyosis and endometrioma.

The computed tomography finding was consistent with acute appendicitis, with a slight increase in diameter (8 mm) and densification of the adjacent fat; however, findings were also compatible with appendiceal endometriosis [5, 8]. However, no images that would be more indicative of endometriosis were observed, such as intraluminal masses [6, 15] or focal nodules [5]. The variability of imaging findings without a pattern, associated with the rare occurrence of this disease, certainly makes it difficult to diagnose appendiceal endometriosis exclusively by imaging methods.

Diagnostic and therapeutic laparoscopy with appendectomy had a primary role in the management of the case and is the first choice of most of the authors studied [7, 8, 14, 15]; it was also our first choice. However, its use needs to be better disseminated in the context of Brazilian public health. In one case [15], there was a conversion to midline infraumbilical laparotomy with *en bloc* appendectomy and segmental resection of the sigmoid by invasion of the appendiceal mass in the terminal ileum and sigmoid colon, which precluded dissection of the structures. In another case [8], conversion to open surgery occurred due to an inability to identify the appendix because of extensive adherence to the abdominal wall. In only one report [16], the initial access route was laparotomy, and the indication was not clear, although it was most likely due to the presence of signs of peritonitis.

Because appendiceal endometriosis may be present in up to 13.2% of cases of deep endometriosis [12, 13], patients undergoing surgical treatment for this condition should be warned of the possible need to perform elective tactical appendectomy.

The anatomopathological analysis revealed two diagnoses, acute suppurative appendicitis with glandular and stromal appendiceal endometriosis, without the presence of dysplasias. This occurrence makes this case even more special, since no other report of inflammatory process with polymorphonuclear infiltrate was found in the literature [6–8, 14–16], and it actually excludes the hypothesis of acute appendicitis.

However, the possibility of a histopathological diagnosis of carcinoid tumours in appendectomy specimens justifies the performance of elective appendectomy in patients with chronic pelvic pain and endometriosis [18]. Conventional surgery can be curative in most of these cases, especially when lesions are smaller than 1 cm. Right hemicolectomy is indicated for tumours larger than 2 cm or with metastatic lymph nodes and mesoapendiceal, peritoneal, or angioinvasive lymph nodes [19].

The patient presented good postoperative evolution with remission of symptoms, and she denied the recurrence of abdominal pain. She was instructed to follow up with gynaecology at an outpatient clinic to investigate deep pelvic endometriosis. If complaints persisted, further investigation with magnetic resonance imaging [5, 6] could offer benefits for the mapping of the disease, especially in the case of extensive intestinal endometriosis. Among the various techniques available for the most severe cases, gynaecological evaluation and hormone therapy [5, 16] could help control symptoms, and elective surgical intervention could even be performed [5].

Appendiceal endometriosis is an uncommon condition, with few reports in the medical literature worldwide. Acute appendicitis secondary to endometriosis is an even less frequent evolution of the disease, with no reports in the last 5 years. Even so, it should be considered in the clinical context of acute abdomen in young women, especially when the initial evaluation is unclear regarding laboratory and imaging findings. The anatomopathological examination of the appendectomy product is mandatory and essential for diagnostic confirmation.
